# Fatal Tight Lacing

**Published:** 1888-11

**Authors:** 


### FATAL TIGHT LACING.

The following from the *London Lancet*, teaches its own moral without comment:

"In our issue of June 25th, we drew attention to the abuse of tight lacing, which possesses, for many wearers of the corset, such a fatal attraction. Shortly before that date, an inquest held upon the body of an elderly female, revealed the fact that death had resulted from this practice. Only a few days ago a nearly similar instance was recorded. In this case a young lady who suffered from fatty infiltration of the heart, died suddenly while dressing hastily after a hearty meal. Here, also, tight lacing played a prominent part in determining the fatal result.

"We had hoped that sensible reflction upon the bad effects of this injurious custom, as illustrated in the history of a former generation, might have impressed upon the would-be-fashionable the obvious teaching of experience. The cases just quoted, however, are probably but a representative minimum of a larger number, which do not come before the coroner's court; and the evil they exemplify, though certainly less general than of yore, still continues to act as a potent cause of ill-health.

"It is hardly necessary to repeat, at length, the causes which render this abuse of the corset so effectively mischievous. Pathologists have a clear perception of all that is implied in its doubtfully graceful discomfort. The displacement of almost all the organs in the chest and abdomen, the compression of several of these upon the heart and great vessels, and the restriction of breathing space which is thus entailed, have in their eyes no beauty, but the sad aspect of feebleness willfully acquired, with the promise of a lifetime as brief as it is practically useless."

